# 888. *In Vitro* Forgiveness of INSTI-Containing Regimens at Drug Concentrations Simulating Variable Adherence

**DOI:** 10.1093/ofid/ofab466.1083

**Published:** 2021-12-04

**Authors:** Rima K Acosta, Andrew Mulato, Michelle L D’Antoni, Stephen R Yant, Tomas Cihlar, Kirsten L White

**Affiliations:** Gilead Sciences, Inc., Foster City, CA

## Abstract

**Background:**

The integrase strand transfer inhibitor (INSTI)-based regimens bictegravir/emtricitabine/tenofovir alafenamide (BIC/FTC/TAF), dolutegravir (DTG)+FTC/TAF, DTG/lamivudine (3TC), and DTG/rilpivirine (RPV) are all used for treatment of HIV-infected patients. Here, relative time to *in vitro* viral breakthrough (VB) and resistance barrier using simulated human drug exposures at either full or suboptimal treatment adherence to each regimen were compared.

**Methods:**

Wild-type HIV-1 (IIIb)-infected MT-2 cells were exposed to the combinations of BIC+FTC+TAF, DTG+FTC+TAF, DTG+3TC, or DTG+RPV for up to 35 days or until VB. Fixed drug concentrations were the human plasma-free adjusted clinical trough concentrations (C_min_) or fixed at simulated C_min_ after missing 1 to 4 consecutive doses (C_min_-1 to -4), with many replicates. Drug resistance was studied by next-generation sequencing at ≥2% frequency.

**Results:**

At drug concentrations corresponding to full adherence and 1 missed dose (C_min_ and C_min_-1), no VB occurred with any regimen (Table). At C_min_-2, only DTG+3TC had VB, with some emergent resistance to both drugs. At C_min_-3, all regimens had VB: by day 12, 100% of DTG+3TC wells had VB; for BIC+FTC+TAF, DTG+FTC+TAF, and DTG+RPV, < 15% of wells had VB which began after day 14. Emergent RT or IN resistance was seen for DTG+RPV and DTG+3TC but not for BIC+FTC+TAF or DTG+FTC+TAF. At C_min_-4, all DTG+3TC and DTG+FTC+TAF wells had VB by day 12, while DTG+RPV had 94% VB by day 25 and BIC+FTC+TAF had 50% VB by day 35. Emergent C_min_-4 drug resistance was seen for all regimens but at differing frequencies; DTG+RPV had the most wells with resistance. Cumulatively, emergent RT and/or IN resistance was found in 1.3% BIC+FTC+TAF, 2.5% DTG+FTC+TAF, 7.9% DTG+3TC, and 8.8% DTG+RPV cultures.

Summary of Forgiveness and Barrier to Resistance of INSTI-Containing Regimens

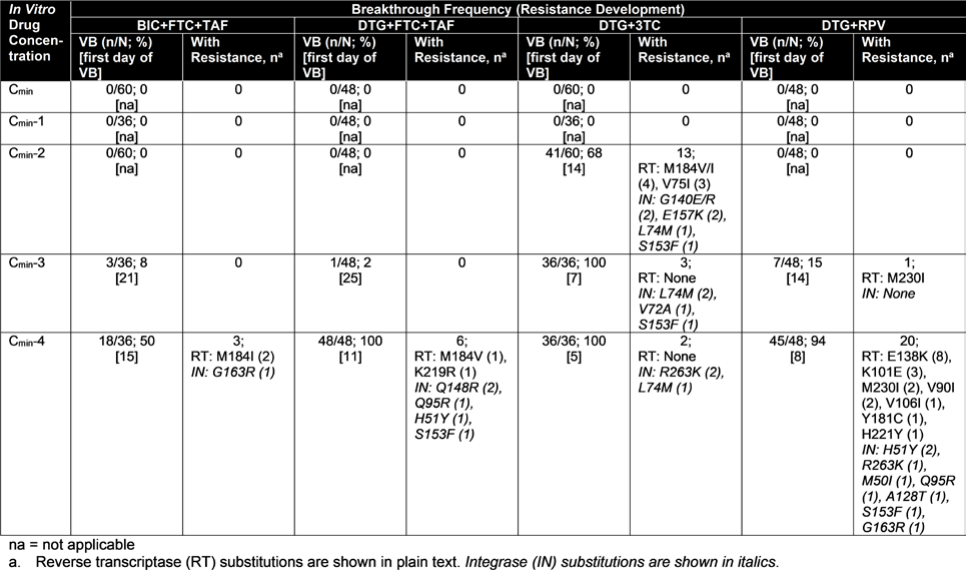

**Conclusion:**

Regimen forgiveness and resistance barrier are important factors in long term treatment. These INSTI-based regimens had high *in vitro* forgiveness and resistance barriers with concentrations simulating high adherence. When multiple missed doses were simulated *in vitro*, BIC+FTC+TAF had the highest forgiveness and barrier to resistance. When compared to DTG+3TC and DTG+FTC+TAF, DTG+RPV had higher forgiveness but lower resistance barrier after several simulated missed doses.

**Disclosures:**

**Rima K. Acosta, BS**, **Gilead Sciences, Inc.** (Employee, Shareholder) **Andrew Mulato, BS, MBA**, **Gilead Sciences, Inc.** (Employee, Shareholder) **Michelle L. D’Antoni, PhD**, **Gilead Sciences** (Employee, Shareholder)**Gilead Sciences, Inc** (Employee, Shareholder) **Stephen R. Yant, PhD**, **Gilead Sciences, Inc.** (Employee, Shareholder) **Tomas Cihlar, PhD**, **Gilead Sciences, Inc.** (Employee, Shareholder) **Kirsten L. White, PhD**, **Gilead Sciences, Inc** (Employee, Shareholder)

